# An individualized reconstructive algorithm for large facial defects after tumor resection in elderly patients: A retrospective case series

**DOI:** 10.1016/j.jpra.2026.09.007

**Published:** 2026-09-12

**Authors:** Xuanda Liu, Zhiguo Yang, Honghong Li

**Affiliations:** Department of Plastic Surgery, Second Affiliated Hospital of Anhui Medical University, Hefei, 230001, Anhui Province, China

**Keywords:** Elderly patients, Large facial defects, Facial neoplasms, Reconstructive algorithm, Surgical flaps, Skin grafting

## Abstract

Reconstruction of large facial defects after tumor resection in elderly patients requires a balance between defect complexity, facial function, and limited physiologic reserve. This retrospective case series included 28 patients aged 65 years or older who were treated between October 2008 and January 2026. A large defect was defined clinically as one unsuitable for primary closure and/or requiring graft or flap reconstruction because of anatomical or functional complexity. Outcomes at the final available follow-up were independently reassessed by two plastic surgeons who had not participated in the index procedures, using de-identified records, postoperative photographs, and a predefined functional checklist. Data were analyzed descriptively. Defect size ranged from 5 × 6 cm to 12 × 16 cm. Full-thickness skin grafts were used in 13 patients, regional or pedicled flaps in 9, and free or bulky myocutaneous flaps in 6. Complete wound closure was achieved in all patients, and all 28 grafts or flaps survived. Five postoperative complications occurred within 30 days: three Clavien-Dindo grade I and two grade II; no grade III-V complications occurred. Median follow-up was 5 months (range, 3–8 months) in the skin-graft group, 10 months (range, 8–24 months) in the regional or pedicled flap group, and 16 months (range, 10–60 months) in the free-flap group. In the 27 definitive/curative-intent cases, no reconstruction failure was documented; the single palliative-intent case achieved stable wound coverage and local symptom control. These findings support an individualized reconstructive strategy based on defect characteristics and operative tolerance.

## Introduction

Facial tumors include benign, premalignant, and malignant lesions arising from the skin or adjacent facial structures.^1^^,^^2^ Because the face is exposed, many lesions are detected early and treated with excision followed by direct closure or local flap repair. In elderly patients, however, delayed presentation may occur because of reduced healthcare-seeking behavior, comorbidities, or reluctance to undergo surgery.^1^^,^^3^ Tumor progression can therefore result in large post-resection defects requiring more complex reconstruction.

Reconstruction in this population must restore function and appearance while limiting operative risk. Defects involving the forehead, periorbital region, cheek, nose, lips, or mandible may compromise eyelid protection, oral competence, speech, facial contour, or tissue coverage. At the same time, hypertension, diabetes mellitus, cardiopulmonary dysfunction, malnutrition, and other systemic conditions may increase the risk of prolonged anesthesia and extensive surgery.^4^^,^^5^ Therefore, reconstructive planning must balance oncologic safety, defect complexity, operative burden, and expected functional and aesthetic outcomes.

Available options include full-thickness skin grafts, local and regional flaps, and free tissue transfer.^2^^,^^6^^,^^7^ Skin grafting is simple and less invasive but is limited in deep or composite defects. Regional flaps provide reliable vascularity and favorable tissue match in selected cases, whereas free flaps remain important for extensive, deep, or through-and-through defects but require greater perioperative support. In elderly patients, the optimal method is determined not by defect size alone but by defect characteristics and patient tolerance.^5^^,^^6^^,^^8^ This study reviewed 28 elderly patients and summarized an individualized reconstructive strategy for large facial defects after tumor resection.

## Materials and methods

### Study design and patient selection

This retrospective case series included patients aged 65 years or older who underwent reconstruction for large facial defects after tumor resection at our institution between October 2008 and January 2026. Reporting followed STROBE recommendations where applicable, and biological sex was recorded from medical records. A large defect was defined clinically as one unsuitable for direct primary closure and/or complex because of size, depth, critical-structure exposure, oral or nasal cavity communication, or involvement of a functionally important facial subunit; no rigid area threshold was used. Eligible patients required reconstruction with a full-thickness skin graft, regional or pedicled flap, or free flap and had complete perioperative and follow-up data. Exclusion criteria were age younger than 65 years, a defect amenable to primary closure, a non-tumor-related defect, incomplete clinical or operative records, insufficient follow-up, or reconstruction outside the institution. Treatment intent was retrospectively classified as definitive/curative or palliative from multidisciplinary assessments, operative records, and documented treatment goals.

A broad search of the hospital electronic medical record system and operative database identified 43 potentially eligible records. Fifteen were excluded: age younger than 65 years (n = 4), defects amenable to primary closure (n = 4), non-tumor-related facial defects (n = 4), incomplete clinical or operative records (n = 1), and insufficient follow-up data (n = 2). The remaining 28 patients were included (Supplementary Fig. S1).

The final cohort comprised 19 male and 9 female patients aged 65 to 95 years. Pathological diagnoses included benign tumors in 6 cases, precancerous lesions in 5, and malignant tumors in 17. Tumor types included sebaceous nevus, melanocytic nevus, neurofibroma, vascular malformation, actinic keratosis, cutaneous horn, basal cell carcinoma, squamous cell carcinoma, metastatic squamous cell carcinoma, and laryngeal carcinoma involving the hypopharynx. Cases of laryngeal carcinoma involving the hypopharynx were included because tumor resection resulted in large cervicofacial defects requiring plastic reconstructive coverage. Post-resection defect size ranged from 5 × 6 cm to 12 × 16 cm. Baseline clinicopathologic and defect characteristics are summarized in Table 1.

**Table 1:** Demographic, Clinicopathological, and Defect Characteristics of the 28 Patients.

Variable	Value
No. of patients	28
Age, years	65–95
Sex, n	Male, 19; female, 9
Tumor type, n	Sebaceous nevus: 1; Melanocytic nevus: 2; Vascular malformation: 2; Neurofibroma: 1; Actinic keratosis: 4; Cutaneous horn: 1; Basal cell carcinoma: 6; Squamous cell carcinoma: 8; Metastatic squamous cell carcinoma: 1; Laryngeal carcinoma invading the hypopharynx: 2
Lesion nature, n	Benign: 6; Premalignant: 5; Malignant: 17
Defect size, cm	5 × 6 to 12 × 16
Primary defect location, n	Forehead: 6; Periorbital region: 4; Cheek/buccal region: 6; Nasal region: 2; Oral commissure/lip: 3; Zygomatic-temporal region: 4; Fronto-orbital region: 3
Major comorbidities, n	Hypertension: 9; Diabetes mellitus: 7; Cardiopulmonary disease: 7; Others: 4
Anesthesia, n	Local: 9; General: 19
Treatment intent, n	Definitive/curative: 27; Palliative: 1

### Preoperative evaluation

All patients underwent preoperative assessment of tumor extent, general medical condition, anesthetic tolerance, and expected reconstructive requirements.^4^^,^^5^ Blood pressure and blood glucose levels were monitored perioperatively. Hematologic, imaging, and pathological findings were reviewed as appropriate, and supportive treatment was provided to optimize systemic condition and organ function. Anesthesiology consultation was performed to assess anesthesia-related risk, estimate operative tolerance, and guide the choice of local or general anesthesia. An individualized perioperative and reconstructive plan was then developed according to the patient’s condition and the expected extent of resection.

### Reconstructive decision-making

Reconstruction was selected according to tumor pathology, defect depth, anatomical location, communication with the oral or nasal cavity, exposed bone or soft-tissue loss, and tolerance for anesthesia and operative duration.^2^^,^^6^^,^^8^ The overall principle was to achieve safe wound closure and restore key facial function while minimizing surgical burden in medically vulnerable elderly patients.

For benign tumors and precancerous lesions confined mainly to the full thickness of the skin, the wound bed after excision usually had adequate vascularity. When the defect was too large for direct closure or simple local flap repair, full-thickness skin grafting under local anesthesia was considered a first-line option, particularly in patients with limited tolerance for prolonged surgery.^6^^,^^7^^,^^9^^,^^10^ This approach was favored because it shortened operative time, reduced perioperative risk, promoted recovery, and avoided excessive flap bulk in selected superficial defects.

For malignant tumors or lesions with suspected malignant potential, extended excision frequently produced deeper defects with exposed bone, soft-tissue loss, or composite deficiency, requiring flap reconstruction.^2^^,^^8^^,^^11^^,^^12^ Random local flaps, subcutaneous pedicled flaps, regional flaps, or free flaps were selected according to reconstructive requirements. Regional pedicled flaps were generally preferred for moderate-volume defects when reliable coverage could be achieved without microsurgery, whereas free flaps were reserved for extensive, deep, or complex defects requiring greater tissue volume or internal lining.

For tumors involving the midface, cheek, mandible, or zygomatic-temporal region, a large ipsilateral platysma myocutaneous flap or lateral cervicofacial flap was used when the periorbital region, oral commissure, and nose were not extensively invaded and when there was no major oral cavity communication or palliative resection was intended.^11^, ^12^, ^13^, ^14^, ^15^ The flap was designed according to defect size and could be modified as an island pedicled flap when greater mobility was required. In selected cases, platysma muscle was included to fill soft-tissue defects, and the donor site was repaired with skin grafting when necessary.

For forehead, zygomatic-temporal, or nasal defects, a forehead flap was selected when thin, well-vascularized tissue with favorable reach was required.^16^, ^17^, ^18^ It was particularly suitable for nasal reconstruction. In selected full-thickness perioral defects with gingival or intraoral exposure, the flap could be folded inward to provide lining while reconstructing the external defect. Staged reconstruction was performed when necessary to restore lip continuity, mucosal lining, and external skin coverage.

For advanced malignant tumors requiring radical resection with partial craniofacial bone removal, orbital exenteration, or removal of adjacent head and neck tumors extending into the cervicofacial region, defects were often deep and extensive. Bulky flap coverage was required to obliterate dead space, protect exposed structures, and reconstruct external and internal surfaces.^8^^,^^19^, ^20^, ^21^ Scapular flaps, pectoralis major myocutaneous flaps, and free latissimus dorsi myocutaneous flaps were used according to defect size, depth, and complexity. For through-and-through defects, part of the flap was folded inward for lining while the remaining tissue provided external coverage.

### Outcome measures and follow-up

Primary outcomes were complete wound closure and graft/flap survival, which referred to survival of the reconstructive tissue rather than patient survival or oncologic cure. Secondary outcomes were postoperative complications, secondary functional revision, and preservation or restoration of major facial functions. Complications during the index admission or within 30 days were retrospectively graded by the Clavien-Dindo classification according to treatment required. Follow-up was calculated from reconstruction to the last documented assessment and summarized by reconstructive group.

At the final available follow-up, outcomes were independently reassessed by an attending plastic surgeon and a senior plastic surgeon, neither of whom participated in the index procedures. They reviewed de-identified records and available photographs using a predefined checklist covering wound closure, graft/flap survival, oral competence, eyelid closure and corneal protection, nasal or oral lining continuity, and secondary functional revision. Disagreements were resolved by consensus. Because FACE-Q and a validated observer-rated aesthetic scale were not prospectively collected, patient satisfaction and aesthetic acceptability were not analyzed.

### Statistical analysis

Because this was a descriptive retrospective case series with a small sample size, continuous variables were summarized as ranges or medians with ranges, and categorical variables were presented as counts. In accordance with journal guidance for small samples, percentages were not reported for data with a total sample size below 50. No comparative inferential statistical analyses were performed.

## Results

### Patient selection and baseline characteristics

Of the 43 potentially eligible patient records assessed, 15 were excluded: age younger than 65 years (n = 4), defects amenable to primary closure (n = 4), non-tumor-related facial defects (n = 4), incomplete clinical or operative records (n = 1), and insufficient follow-up data (n = 2). The remaining 28 patients were included in the final analysis (Supplementary Fig. S1). Baseline clinicopathologic and defect characteristics are presented in Table 1.

### Wound closure, graft/flap survival, and complications

Complete wound closure was achieved in all 28 patients. Full-thickness skin grafts were used in 13 patients, regional or pedicled flaps in 9, and free or bulky myocutaneous flap reconstruction in 6. All 28 grafts or flaps survived; this endpoint refers specifically to survival of the reconstructive tissue and not to patient survival or oncologic cure. Five patients developed postoperative complications during the index admission or within 30 days: 2 in the full-thickness skin-graft group, 2 in the regional or pedicled flap group, and 1 in the free-flap group. Three complications were Clavien-Dindo grade I and two were grade II. No grade III-V complications occurred, and no complication resulted in loss of the reconstructive result or required major salvage reconstruction. Reconstructive methods, complications, and subgroup-specific follow-up are summarized in Table 2.

**Table 2:** Reconstructive Method, Graft/Flap Survival, Complications, and Follow-up.

Reconstructive method	No. of patients, n	Graft/flap survival, n/N	Patients with 30-day complications, n	Follow-up duration, months, median (range)
Full-thickness skin graft	13	13/13	2	5 (3–8)
Regional or pedicled flap	9	9/9	2	10 (8–24)
Free or bulky myocutaneous flap reconstruction	6	6/6	1	16 (10–60)
Total	28	28/28	5	—

Complications during the index admission or within 30 days were graded retrospectively using the Clavien-Dindo classification: grade I, n = 3; grade II, n = 2; no grade III-V. Patients were grouped by the primary reconstructive method. Follow-up was measured from reconstruction to the last documented clinical assessment.

### Reconstructive and functional outcomes

At the final available follow-up, independent reassessment documented stable wound coverage without reconstruction failure. In perioral or lip-related defects, oral continuity and competence were preserved or restored. In periorbital or upper facial defects, eyelid closure, corneal protection, and wound stability were maintained without major functional impairment. When reconstruction of the nasal or oral lining was required, continuity was restored using folded or composite flap techniques. No unplanned major secondary functional revision was documented during the available follow-up; planned staged procedures were considered part of the original reconstructive strategy.

### Subgroup follow-up and outcomes by treatment intent

Median follow-up was 5 months (range, 3–8 months) in the full-thickness skin-graft group, 10 months (range, 8–24 months) in the regional or pedicled flap group, and 16 months (range, 10–60 months) in the free-flap group. No reconstruction failure was documented during the available follow-up. Treatment intent was definitive/curative in 27 patients and palliative in 1. In the definitive/curative-intent group, complete wound closure was achieved in all patients and no reconstruction failure was documented. Patients with malignant tumors received additional oncologic treatment when indicated. In the single palliative-intent case, stable wound coverage and local wound control were achieved, with improvement in infection-related symptoms and malodor. The study was not designed to evaluate long-term cancer-specific survival, and oncologic cure is not claimed.

### Representative clinical cases

**Fig. 1:**
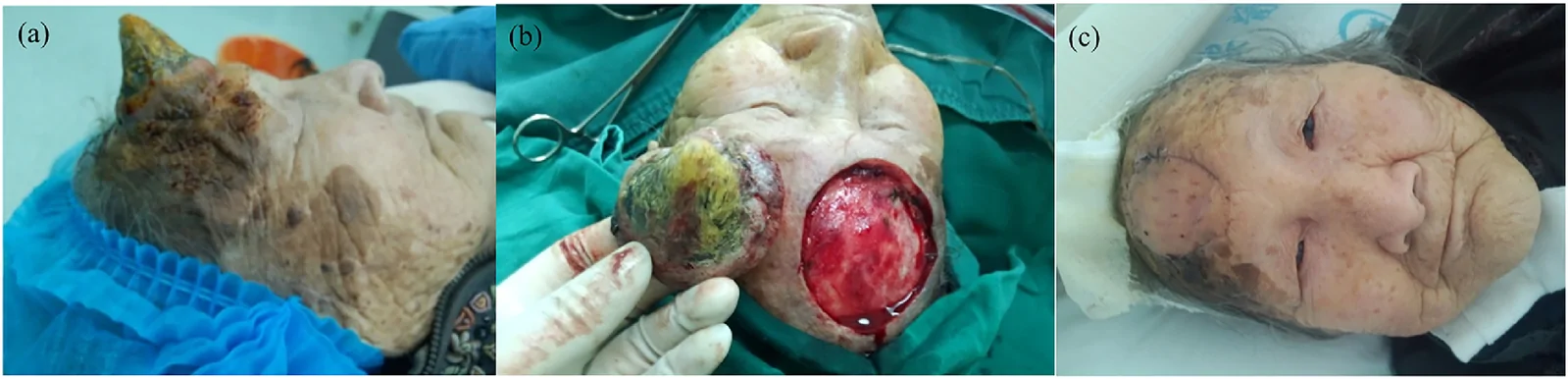
Representative case 1.

Representative Case 1. A 95-year-old woman presented with a horn-like forehead lesion that had enlarged over >10 years. She also had chronic bronchitis, emphysema, and cor pulmonale. Extended excision was performed under local anesthesia with electrocardiographic monitoring, producing a post-resection defect of approximately 5 × 6 cm that was unsuitable for primary closure. The wound was reconstructed with a full-thickness skin graft. Pathology revealed keratoacanthoma, and the patient was discharged 1 week after suture removal (Fig. 1a-c).

a Preoperative appearance. b Intraoperative wound defect. c Postoperative appearance at 1 week.

**Fig. 2:**
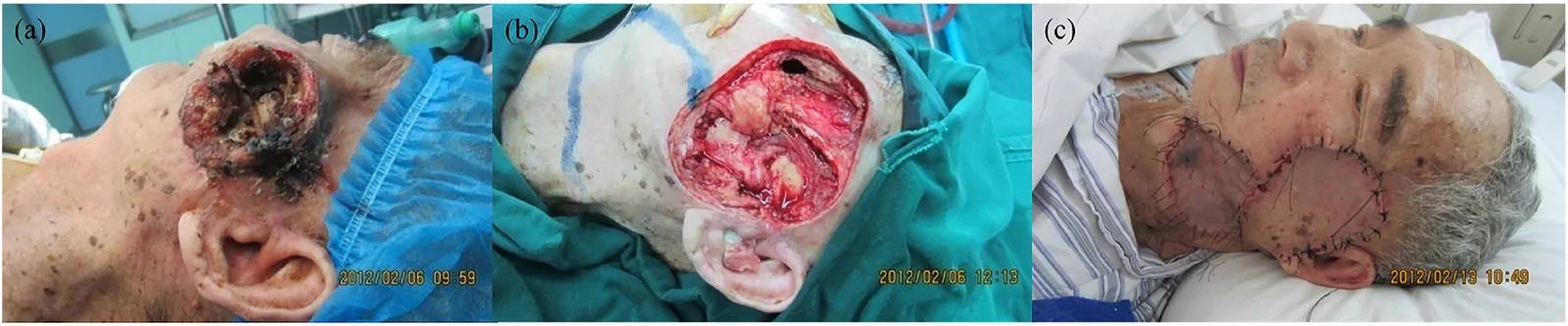
Representative case 2.

Representative Case 2. An 87-year-old man presented with recurrent squamous cell carcinoma of the zygomatic and buccal region, accompanied by infection, purulent discharge, and malodor. Magnetic resonance imaging showed deep tissue invasion. Because the family requested palliative treatment and declined high-risk reconstruction, extended tumor resection and cervical lymph node dissection were performed under general anesthesia. A 10 × 8 cm defect with exposed bone was reconstructed with a platysma myocutaneous flap, and the donor site was repaired with skin grafting. The patient was discharged 10 days postoperatively (Fig. 2a-c).

a Preoperative appearance. b Intraoperative wound defect and design of the platysma myocutaneous flap. c Postoperative appearance at 1 week.

**Fig. 3:**
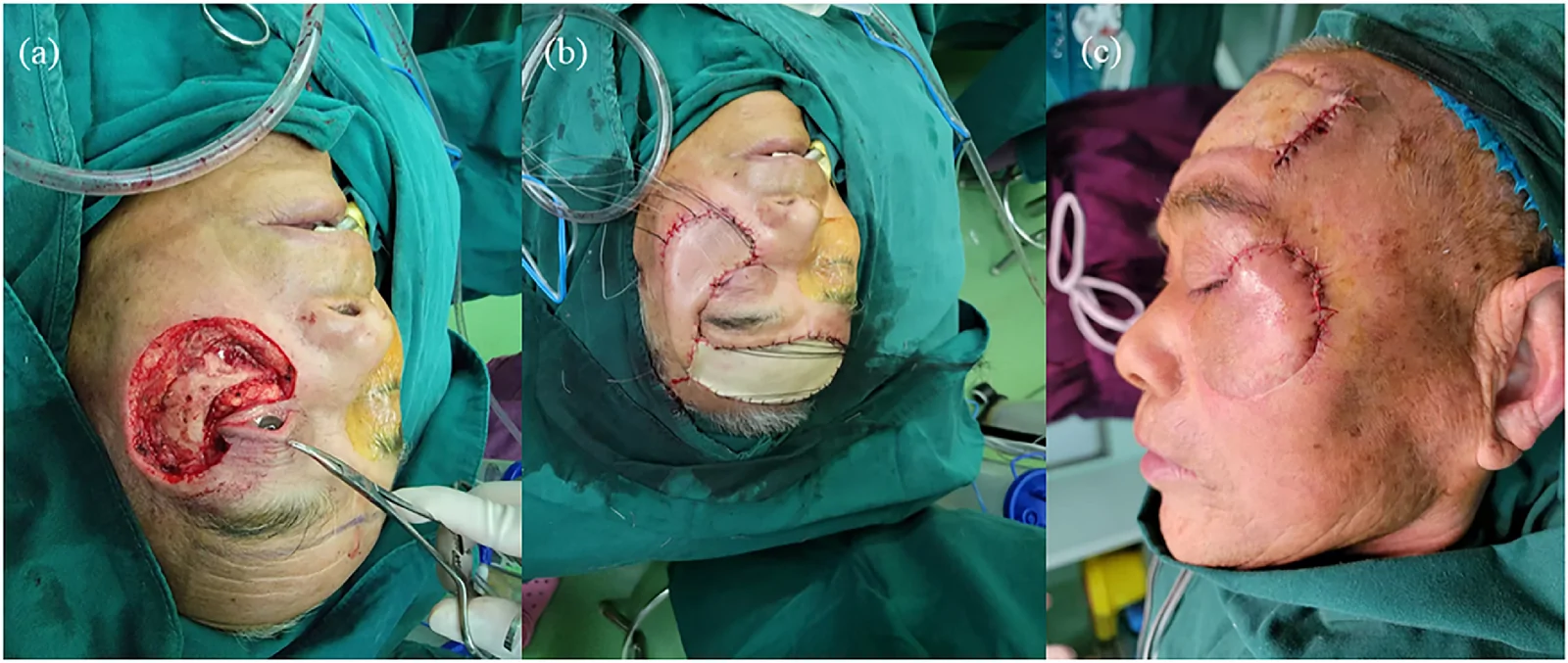
Representative case 3.

Representative Case 3. An 85-year-old man presented with a 3 × 2 cm ulcerative lesion of the left lower eyelid; this measurement referred to the preoperative lesion rather than the reconstructive defect. Mohs micrographic surgery confirmed basosquamous carcinoma invading subcutaneous fat. After extended excision, the post-resection periocular defect measured approximately 5 × 6 cm and included exposed orbital bone. It was reconstructed with a folded island forehead flap, and the donor site was covered with an abdominal full-thickness skin graft. Three weeks later, planned pedicle division, flap refinement, and lateral canthoplasty were performed. The patient was discharged 7 days after the second-stage operation (Fig. 3a-c).

a Intraoperative wound defect. b Design of the forehead island pedicled flap. c Second-stage pedicle division, flap refinement, and lateral canthoplasty.

**Fig. 4:**
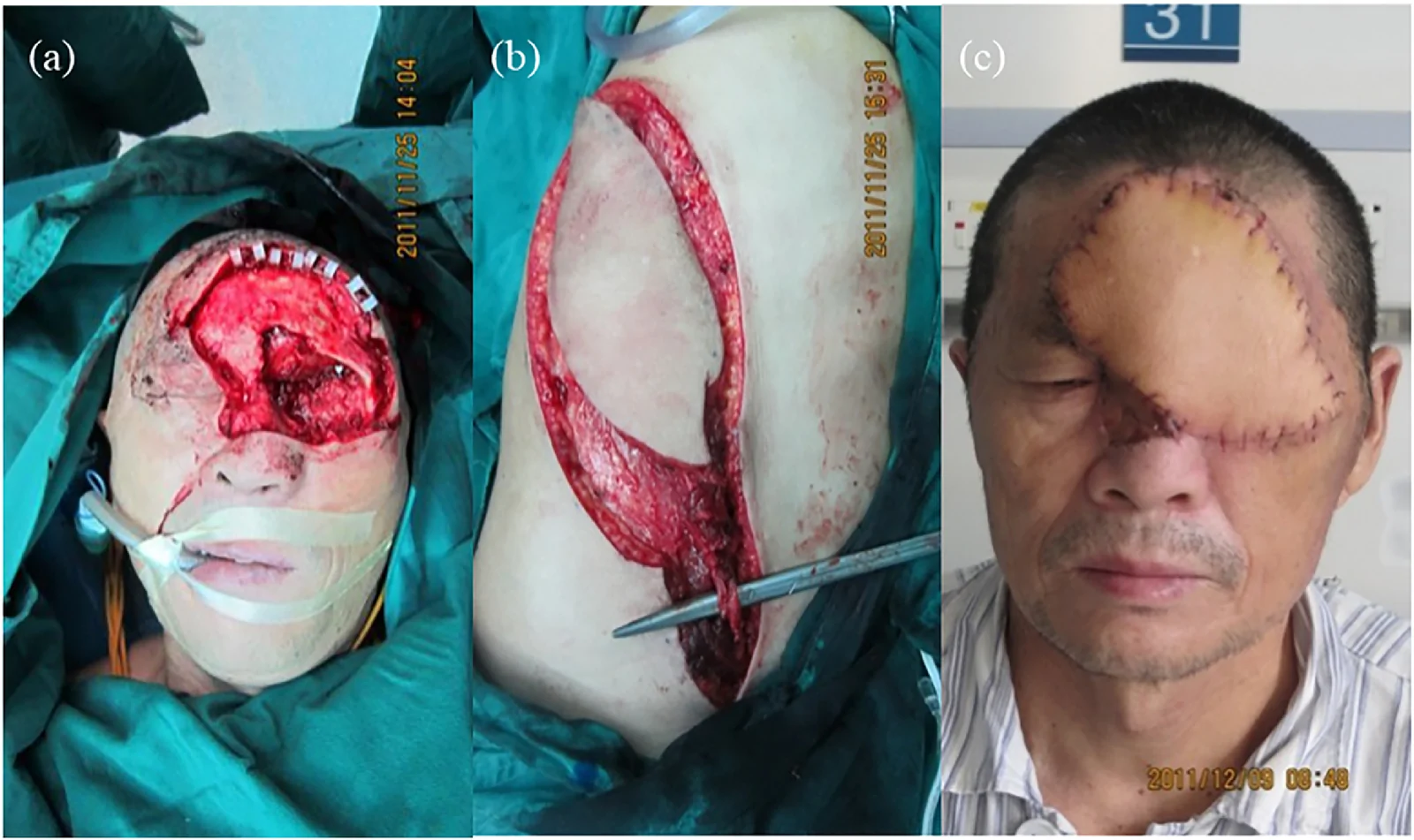
Representative case 4.

Representative Case 4. A 68-year-old man underwent radical resection by ophthalmology and otolaryngology teams for a tumor involving the frontal sinus and orbit, leaving an approximately 12 × 14 cm defect with a depth of about 5 cm. A latissimus dorsi myocutaneous flap was harvested from the left thoracodorsal region, and the thoracodorsal vessels were anastomosed to the left superficial temporal vessels. The donor site was closed primarily, and the flap survived completely. Pathology suggested a spindle cell tumor, with inflammatory myofibroblastic tumor considered possible, and postoperative radiotherapy was administered (Fig. 4a-c).

a Frontal view of the intraoperative wound defect. b Design and harvest of the latissimus dorsi free flap. c Postoperative appearance at 2 weeks.

## Discussion

This study suggests that large facial defects after tumor resection in elderly patients can be effectively managed using an individualized reconstructive strategy that balances defect complexity, tumor behavior, and operative tolerance. Full-thickness skin grafts, regional flaps, and selected free flaps each had distinct roles. The central issue in elderly patients is not simply wound closure, but achievement of durable coverage and preservation of essential facial function while limiting perioperative burden.^2^^,^^3^^,^^5^^,^^8^

Full-thickness skin grafting was useful in selected elderly patients with superficial defects and well-vascularized wound beds. Although graft-based reconstruction can be limited by contour depression, contracture, and color or texture mismatch,^6^^,^^7^^,^^9^ it offers important advantages in elderly patients, including shorter operative time, lower anesthetic stress, feasibility under local anesthesia, faster recovery, and avoidance of bulky tissue transfer. In this series, grafting was used when the defect was superficial, the wound bed was adequate, and prolonged surgery was undesirable. For such defects, grafting may therefore be a rational first-line option rather than a fallback technique.^6^^,^^7^^,^^10^

Regional and pedicled flaps provided an important intermediate option. For moderate-volume defects requiring vascularized tissue but not necessarily microsurgical transfer, platysma flaps, lateral cervicofacial flaps, and forehead flaps provided reliable coverage with acceptable tissue match and lower operative complexity.^11^, ^12^, ^13^, ^14^, ^15^, ^16^ Island design or staged transfer increased reach and versatility in selected cases. These flaps are particularly valuable when the defect is too deep or extensive for grafting alone but the patient’s condition or reconstructive goal does not justify a prolonged microsurgical procedure.

Free flaps and bulky myocutaneous flaps remained indispensable for advanced, deep, or composite defects.^8^^,^^19^, ^20^, ^21^ When resection resulted in exposed bone, dead space, cavity communication, or through-and-through tissue loss, simple coverage was insufficient. Adequate tissue volume, reliable vascularity, and sometimes internal lining were required. Chronological age alone should not be an absolute contraindication to microsurgery, but physiologic reserve, anesthetic tolerance, nutritional status, vascular condition, and reconstructive goals must be considered.^8^^,^^19^^,^^20^ In palliative cases, the most appropriate reconstruction may be the one that safely achieves wound control and symptom relief rather than maximal technical complexity.

Preoperative optimization and multidisciplinary planning were also important.^4^^,^^5^^,^^8^ Hypertension, diabetes mellitus, cardiopulmonary dysfunction, infection, and malnutrition can increase surgical risk in elderly patients. In this series, favorable outcomes were supported by perioperative assessment, optimization of systemic conditions, anesthetic evaluation, and individualized planning. Thus, reconstructive success depended not only on flap selection but also on appropriate patient selection and perioperative management.

Based on this experience, a practical individualized reconstructive algorithm is proposed in Table 3. Superficial skin-confined defects with adequate wound beds and limited operative tolerance are suited to full-thickness skin grafting.^6^^,^^7^^,^^10^ Moderate-volume defects without major cavity communication can often be reconstructed with regional or pedicled flaps.^11^, ^12^, ^13^, ^14^, ^15^, ^16^ Extensive, deep, composite, or through-and-through defects generally require bulky flap coverage or free tissue transfer.^8^^,^^19^, ^20^, ^21^ This framework is intended to support, not replace, surgical judgment in elderly patients for whom safety, durable wound closure, and preservation of major facial function are critical.

**Table 3:** Proposed Individualized Reconstructive Algorithm for Elderly Patients With Large Facial Defects.

Defect/Patient Scenario	Key Features	Preferred Reconstruction	Rationale
Superficial skin-confined defect	Adequate wound bed; no major soft-tissue loss; limited tolerance for prolonged surgery	Full-thickness skin graft	Shorter operative time, lower perioperative burden, and feasibility under local anesthesia
Moderate soft-tissue defect	Requires vascularized tissue; no major cavity communication; no need for bulky tissue volume	Regional or pedicled flap	Reliable blood supply, adequate soft-tissue replacement, and avoidance of microsurgery
Midfacial, buccal, mandibular, or zygomatic-temporal defect	Moderate-volume defect; selected cases without major oral cavity communication	Platysma myocutaneous flap or lateral cervicofacial flap	Good flap reach, reliable vascularity, and limited operative burden
Forehead, nasal, or perioral defect	Thin and pliable tissue needed; staged reconstruction or lining reconstruction may be required	Forehead flap	Favorable reach and pliability; suitable for nasal and selected lip defects
Deep or composite defect	Exposed bone, dead space, extensive tissue loss, or internal lining requirement	Free or bulky myocutaneous flap reconstruction	Provides sufficient tissue volume and durable coverage
Medically fragile elderly patient	High anesthetic risk or major systemic comorbidities	Least invasive effective reconstruction when oncologically appropriate	Prioritizes safety, wound closure, and reduction of surgical stress

The proposed algorithm is intended to guide reconstructive decision-making and should be individualized according to tumor extent, defect characteristics, and patient tolerance.

### Limitations

This study has limitations. Its retrospective, single-center design and small sample create potential selection bias, and the defects were heterogeneous in pathology, location, treatment intent, and complexity. Functional outcomes were reassessed from available records and photographs rather than collected prospectively with a validated instrument. FACE-Q and a validated aesthetic scale were unavailable, so patient satisfaction and aesthetic acceptability could not be evaluated. Follow-up differed among groups and was relatively short after skin grafting, which may limit detection of late complications. The single palliative case cannot support broad conclusions, and cancer-specific survival or oncologic cure was not assessed. The proposed algorithm requires validation in larger multicenter studies.

## Conclusion

Large facial defects after tumor resection in elderly patients should be reconstructed according to defect depth, location, tumor biology, treatment intent, and operative tolerance. Full-thickness skin grafts, regional flaps, and selected free flaps each have distinct roles within a practical reconstructive algorithm. With careful preoperative optimization and rational method selection, durable wound closure, graft or flap survival, and preservation of major facial function can be achieved while limiting perioperative risk.

## Declaration of generative AI and AI-assisted technologies in the manuscript preparation process

During the preparation of this work, the authors used ChatGPT (OpenAI) for language editing and manuscript formatting assistance. After using this tool, the authors reviewed and edited the content as needed and take full responsibility for the content of the manuscript.

## Ethical approval

This retrospective study was approved by the Medical Research Ethics Committee of the Second Affiliated Hospital of Anhui Medical University (Approval No YX2025–346). All procedures involving human participants were performed in accordance with the ethical standards of the institutional and/or national research committee and with the 1964 Helsinki Declaration and its later amendments or comparable ethical standards.

## Consent to participate

For this type of retrospective study, formal consent was not required.

## Consent for publication

Written informed consent for publication of identifiable clinical images was obtained from the patients or their legal guardians where applicable.

## Funding

This research did not receive any specific grant from funding agencies in the public, commercial, or not-for-profit sectors.

## CRediT authorship contribution statement

**Xuanda Liu:** Conceptualization, Methodology, Investigation, Formal analysis, Writing – original draft. **Zhiguo Yang:** Conceptualization, Methodology, Investigation, Formal analysis, Writing – original draft. **Honghong Li:** Supervision, Writing – review & editing.

## Declaration of competing interest

The authors declare that they have no conflict of interest.

## Data Availability

The datasets generated and/or analyzed during the current study are available from the corresponding author on reasonable request, subject to institutional and ethical restrictions.

## Appendix Supplementary materials

Supplementary Figure Legend Supplementary Fig. S1. STROBE patient screening and inclusion flow diagram.
