# Supplementary material for: An individualized reconstructive algorithm for large facial defects after tumor resection in elderly patients: A retrospective case series
Source: JPRAS Open. 2026 Sep 12;52:433–40. doi: 10.1016/j.jpra.2026.09.007 (PMC13599561; doi:10.1016/j.jpra.2026.09.007)
Supplement: Supplementary file 1 [file mmc2.pdf]

Supplementary Fig. S1. STROBE Patient Screening and Inclusion Flow Diagram

Potentially eligible patient records identified from the hospital electronic medical record system and operative database between October 2008 and January 2026
(n = 43)

↓

Patient records assessed against the predefined eligibility criteria
(n = 43)

↓

Patients excluded (n = 15)

- Age younger than 65 years (n = 4)
- Defects amenable to primary closure (n = 4)
- Non-tumor-related facial defects (n = 4)
- Incomplete clinical or operative records (n = 1)
- Insufficient follow-up data (n = 2)

↓

Patients included in final analysis
(n = 28)
