# Supplementary material for: An individualized reconstructive algorithm for large facial defects after tumor resection in elderly patients: A retrospective case series
Source: JPRAS Open. 2026 Sep 12;52:433–40. doi: 10.1016/j.jpra.2026.09.007 (PMC13599561; doi:10.1016/j.jpra.2026.09.007)
Supplement: Supplementary file 2 [file mmc1.docx]

STROBE Statement—checklist of items that should be included in reports of observational studies

|  | Item No. | Recommendation | Page  No. | Relevant text from manuscript |
| --- | --- | --- | --- | --- |
| **Title and abstract** | 1 | (*a*) Indicate the study’s design with a commonly used term in the title or the abstract | 4 | The manuscript title identifies the design as “A Retrospective Case Series,” and the Summary states that 28 patients aged 65 years or older were included. |
|  |  | (*b*) Provide in the abstract an informative and balanced summary of what was done and what was found | 4 | The Summary reports the clinical definition of a large defect, independent outcome reassessment, reconstructive methods, 30-day Clavien-Dindo complications, subgroup-specific follow-up, treatment intent, and the principal reconstructive findings. |
| Introduction | | | |  |
| Background/rationale | 2 | Explain the scientific background and rationale for the investigation being reported | 5 | The Introduction explains why reconstruction of large post-resection facial defects in elderly patients must balance oncologic safety, defect complexity, preservation of facial function, and operative tolerance. |
| Objectives | 3 | State specific objectives, including any prespecified hypotheses | 5 | The objective was to review 28 elderly patients and summarize an individualized reconstructive strategy for large facial defects after tumor resection. |
| Methods | | | |  |
| Study design | 4 | Present key elements of study design early in the paper | 6 | The Study Design and Patient Selection section identifies the work as a retrospective case series and presents the eligibility framework at the beginning of the Methods. |
| Setting | 5 | Describe the setting, locations, and relevant dates, including periods of recruitment, exposure, follow-up, and data collection | 6, 9-11 | The study was conducted at the authors’ institution between October 2008 and January 2026. Follow-up was calculated from reconstruction to the last documented assessment and was summarized separately by reconstructive group. |
| Participants | 6 | (*a*) *Cohort study*—Give the eligibility criteria, and the sources and methods of selection of participants. Describe methods of follow-up  *Case-control study*—Give the eligibility criteria, and the sources and methods of case ascertainment and control selection. Give the rationale for the choice of cases and controls  *Cross-sectional study*—Give the eligibility criteria, and the sources and methods of selection of participants | 6, 9-10 | Eligibility criteria, data sources used for case identification, the screening and exclusion process, and the method used to calculate follow-up are described in the Study Design and Patient Selection and Outcome Measures and Follow-up sections. |
|  |  | (*b*) *Cohort study*—For matched studies, give matching criteria and number of exposed and unexposed  *Case-control study*—For matched studies, give matching criteria and the number of controls per case | N/A | Not applicable. This was a descriptive retrospective case series without matched exposed/unexposed groups or controls. |
| Variables | 7 | Clearly define all outcomes, exposures, predictors, potential confounders, and effect modifiers. Give diagnostic criteria, if applicable | 9-10 | Primary outcomes were complete wound closure and graft/flap survival. Secondary outcomes were 30-day postoperative complications, secondary functional revision, and preservation or restoration of major facial functions. Treatment intent and the meaning of graft/flap survival were explicitly defined. |
| Data sources/ measurement | 8* | For each variable of interest, give sources of data and details of methods of assessment (measurement). Describe comparability of assessment methods if there is more than one group | 6-10 | Electronic medical records, the operative database, pathology, imaging, operative records, follow-up documentation, and available postoperative photographs were reviewed. At the final available follow-up, two plastic surgeons who had not participated in the index procedures independently reassessed de-identified records and photographs using a predefined functional checklist; disagreements were resolved by consensus. |
| Bias | 9 | Describe any efforts to address potential sources of bias | 6, 9-10, 15 | Eligibility criteria and the screening process were predefined and reported. Outcome reassessment was performed independently by two surgeons who had not participated in the index procedures, using de-identified materials and a predefined checklist. Remaining risks of selection and measurement bias are acknowledged in the Limitations section. |
| Study size | 10 | Explain how the study size was arrived at | 6, 10; Suppl. S1 | A broad search identified 43 potentially eligible records. Fifteen records were excluded for prespecified reasons, leaving 28 patients in the final analysis. The screening process is shown in Supplementary Fig. S1. |

Continued on next page

| Quantitative variables | 11 | Explain how quantitative variables were handled in the analyses. If applicable, describe which groupings were chosen and why | 10 | Continuous variables were summarized as ranges or medians with ranges, and categorical variables were presented as counts. Follow-up was grouped according to the primary reconstructive method. |
| --- | --- | --- | --- | --- |
| Statistical methods | 12 | (*a*) Describe all statistical methods, including those used to control for confounding | 10 | The analysis was descriptive. No comparative inferential analyses or confounder-adjusted estimates were performed. |
|  |  | (*b*) Describe any methods used to examine subgroups and interactions | 6, 9-11, 20-21 | Descriptive subgroup summaries were prespecified for reconstructive method and treatment intent. Follow-up was summarized separately for the skin-graft, regional/pedicled-flap, and free-flap groups; outcomes were also reported separately for definitive/curative and palliative intent. No interaction testing was performed. |
|  |  | (*c*) Explain how missing data were addressed | 6, 10 | Records with incomplete clinical or operative information or insufficient follow-up were excluded during screening. The 28 analyzed patients met the requirement for complete perioperative and follow-up data. |
|  |  | (*d*) *Cohort study*—If applicable, explain how loss to follow-up was addressed  *Case-control study*—If applicable, explain how matching of cases and controls was addressed  *Cross-sectional study*—If applicable, describe analytical methods taking account of sampling strategy | 6, 9-11; Suppl. S1 | Two records were excluded because of insufficient follow-up. For included patients, follow-up extended from reconstruction to the last documented clinical assessment and was reported by reconstructive subgroup. |
|  |  | (*e*) Describe any sensitivity analyses | N/A | Not applicable. No sensitivity analyses were performed. |
| Results | | | | |
| Participants | 13* | (a) Report numbers of individuals at each stage of study—eg numbers potentially eligible, examined for eligibility, confirmed eligible, included in the study, completing follow-up, and analysed | 6, 10; Suppl. S1 | Forty-three potentially eligible records were assessed. Fifteen were excluded, and the remaining 28 patients underwent reconstruction and were included in the final analysis. The numbers at each stage are reported in the manuscript and Supplementary Fig. S1. |
|  |  | (b) Give reasons for non-participation at each stage | 6, 10; Suppl. S1 | Reasons for exclusion were age younger than 65 years (n = 4), a defect amenable to primary closure (n = 4), a non-tumor-related defect (n = 4), incomplete clinical or operative records (n = 1), and insufficient follow-up data (n = 2). |
|  |  | (c) Consider use of a flow diagram | 6, 10; Suppl. S1 | A STROBE patient screening and inclusion flow diagram is provided as Supplementary Fig. S1. |
| Descriptive data | 14* | (a) Give characteristics of study participants (eg demographic, clinical, social) and information on exposures and potential confounders | 6-7, 20 | Demographic, clinicopathological, defect, comorbidity, anesthesia, and treatment-intent characteristics of the 28 patients are summarized in Table 1. |
|  |  | (b) Indicate number of participants with missing data for each variable of interest | 6, 10 | The final analysis required complete perioperative and follow-up data. One record with incomplete clinical or operative information and two records with insufficient follow-up were excluded during screening; no missing-data imputation was performed. |
|  |  | (c) *Cohort study*—Summarise follow-up time (eg, average and total amount) | 4, 11, 20-21 | Median follow-up was 5 months (range, 3-8 months) in the full-thickness skin-graft group, 10 months (range, 8-24 months) in the regional or pedicled flap group, and 16 months (range, 10-60 months) in the free-flap group. |
| Outcome data | 15* | *Cohort study*—Report numbers of outcome events or summary measures over time | 10-11, 20-21 | Complete wound closure, graft/flap survival, 30-day complications, functional outcomes, subgroup-specific follow-up, and outcomes by treatment intent are reported in the Results section and Table 2. |
|  |  | *Case-control study—*Report numbers in each exposure category, or summary measures of exposure | N/A | Not applicable. This was not a case-control study. |
|  |  | *Cross-sectional study—*Report numbers of outcome events or summary measures | N/A | Not applicable. This was not a cross-sectional study. |
| Main results | 16 | (*a*) Give unadjusted estimates and, if applicable, confounder-adjusted estimates and their precision (eg, 95% confidence interval). Make clear which confounders were adjusted for and why they were included | 10-11 | Complete wound closure was achieved in all 28 patients, and all 28 grafts or flaps survived. Five patients developed 30-day complications: three Clavien-Dindo grade I and two grade II; no grade III-V complications occurred. No comparative or adjusted estimates were calculated. |
|  |  | (*b*) Report category boundaries when continuous variables were categorized | N/A | Not applicable. Continuous variables were not categorized for inferential analysis. |
|  |  | (*c*) If relevant, consider translating estimates of relative risk into absolute risk for a meaningful time period | N/A | Not applicable. No relative risk or comparative risk estimates were calculated. |

Continued on next page

| Other analyses | 17 | Report other analyses done—eg analyses of subgroups and interactions, and sensitivity analyses | 11, 20-21 | Descriptive analyses were reported by reconstructive subgroup and treatment intent. Follow-up differed by reconstructive method, and outcomes were reported separately for 27 definitive/curative-intent cases and the single palliative-intent case. No interaction or sensitivity analyses were performed. |
| --- | --- | --- | --- | --- |
| Discussion | | | | |
| Key results | 18 | Summarise key results with reference to study objectives | 13-15 | The Discussion summarizes the roles of full-thickness skin grafts, regional or pedicled flaps, and free or bulky myocutaneous flaps within an individualized strategy focused on durable coverage, preservation of major facial function, and limitation of perioperative burden. |
| Limitations | 19 | Discuss limitations of the study, taking into account sources of potential bias or imprecision. Discuss both direction and magnitude of any potential bias | 15 | The Limitations section discusses the retrospective single-center design, small sample, heterogeneous defects and treatment intent, possible selection bias, retrospective outcome reassessment without validated patient-reported or aesthetic instruments, unequal and relatively short subgroup follow-up, the single palliative case, and lack of cancer-specific survival assessment. |
| Interpretation | 20 | Give a cautious overall interpretation of results considering objectives, limitations, multiplicity of analyses, results from similar studies, and other relevant evidence | 13-15 | The Discussion interprets the findings cautiously in relation to defect characteristics, reconstructive requirements, physiologic reserve, treatment intent, prior evidence, and the study’s methodological limitations. |
| Generalisability | 21 | Discuss the generalisability (external validity) of the study results | 15 | The proposed algorithm was derived from a small, heterogeneous, single-center series and therefore requires validation in larger multicenter studies before broader application. |
| Other information | |  | | |
| Funding | 22 | Give the source of funding and the role of the funders for the present study and, if applicable, for the original study on which the present article is based | 16 | The authors state that the study received no specific grant from public, commercial, or not-for-profit funding agencies; therefore, no funder had a role in the study. |

*Give information separately for cases and controls in case-control studies and, if applicable, for exposed and unexposed groups in cohort and cross-sectional studies.

**Note:** An Explanation and Elaboration article discusses each checklist item and gives methodological background and published examples of transparent reporting. The STROBE checklist is best used in conjunction with this article (freely available on the Web sites of PLoS Medicine at http://www.plosmedicine.org/, Annals of Internal Medicine at http://www.annals.org/, and Epidemiology at http://www.epidem.com/). Information on the STROBE Initiative is available at www.strobe-statement.org.
